# A Systematic Review of Parental Involvement in Cognitive Behavioural Therapy for Adolescent Anxiety Disorders

**DOI:** 10.1007/s10567-020-00324-2

**Published:** 2020-08-30

**Authors:** Jessica Louise Cardy, Polly Waite, Francesca Cocks, Cathy Creswell

**Affiliations:** 1 Oxford Institute of Clinical Psychology, University of Oxford, Oxford, UK; 2 School of Psychology & Clinical Language Sciences, University of Reading, Whiteknights, Reading, RG6 6AL UK; 3 Berkshire Eating Disorders Service, St Mark’s Hospital, Berkshire, UK; 4 Departments of Experimental Psychology and Department of Psychiatry, University of Oxford, Oxford, UK

**Keywords:** Adolescent, Anxiety disorders, Parental involvement, Cognitive behavioural therapy

## Abstract

Anxiety disorders are common among adolescents and lead to poor long-term outcomes. Cognitive Behavioural Therapy (CBT) is an evidenced-based intervention for adolescent anxiety disorders, but little is known about whether and how parents should be involved. This systematic review evaluated how parents have been involved and associated treatment outcomes in studies of CBT for adolescent anxiety disorders. Electronic systematic searches were conducted in PsycINFO, Embase, CINAHL, Medline, AMED databases, to identify studies investigating CBT for adolescent anxiety disorder(s) that included parents in treatment. Twenty-three papers were identified. Parents were involved in treatment in a number of different ways: by attending separate parent sessions, joint parent–adolescent sessions, or both, or through provision of a workbook while attending some adolescent sessions. Content varied but was most typically aimed at the parent developing an understanding of core CBT components and skills to help them manage their adolescent’s anxiety and avoidance. Treatment outcomes indicate that CBT with parental involvement is an effective intervention for adolescent anxiety disorders; however, it is not possible to draw conclusions regarding whether parental involvement (generally or in any particular form) *enhances* treatment outcomes. Poor reporting and methodological issues also limit the conclusions. Further research is required to identify whether there are particular types of parental involvement in CBT that bring clinical benefits to adolescents with anxiety disorders generally, as well as in particular circumstances.

**Electronic supplementary material:**

The online version of this article (10.1007/s10567-020-00324-2) contains supplementary material, which is available to authorized users.

## Introduction

Anxiety disorders are highly prevalent during adolescence; with, for example, 7.9% of 11- to 16-year olds and 13.1% of 17- to 19-year olds identified as having an anxiety disorder in a recent survey in England (Vizard et al. 2018). This is of serious consequence, as adolescent anxiety disorders predict impaired long-term outcomes, including compromised coping skills, work adjustment, life satisfaction, and interpersonal relationships (Essau et al. 2014).

Psychological intervention, specifically Cognitive Behavioural Therapy (CBT), is recommended as a first line intervention for anxiety disorders in children and young people, in preference to pharmacological treatment (World Health Organization, 2015; National Institute for Health and Care Excellence; NICE; 2013), with average remission rates of 59% post-CBT (James et al. 2013). However, treatment studies have typically included children and young people from broad age ranges (Hill et al. 2016), leaving adolescents with anxiety disorders as an under-researched population (Kendall and Ollendick 2005). This is despite there being clear differences in the characteristics of anxiety disorders in adolescents compared to children, including more severe symptoms, comorbid mood disorders, and difficulties attending school (Weems 2008; Waite and Creswell 2015). Furthermore, a large randomised-controlled trial reported poorer remission rates from CBT for adolescents compared to children (Ginsburg et al. 2011). As such, further research is clearly needed to identify how to optimize treatments for adolescents with anxiety disorders.

One aspect of treatment that is likely to need to differ between children and adolescents with anxiety disorders is how parents are involved. This is due to adolescents’ normative drive for increased autonomy (Erikson 1968), their increased capacity for abstract, hypothetical reasoning (Piaget and Inhelder 1969), self-awareness and self-reflection (Blakemore and Choudhury 2006), and because patterns of parent–child interactions in the context of anxiety appear to differ between children and adolescents (Waite and Creswell 2015). Parental factors associated with adolescent anxiety disorders specifically include perceived parental control, parental modelling/reinforcement of anxious behaviours (Waite et al. 2014), and low parental warmth (Waite and Creswell, 2015). However, there remains a lack of clarity about whether and how parents should be involved in CBT for adolescent anxiety disorders and interventions have differed with respect to the number, format, and content of parent sessions (Barmish and Kendall 2005).

Where previous reviews have considered outcomes in relation to parent involvement (e.g. Zhou, Zhang, Furukawa, Cuijpers et al. 2019; Reynolds et al. 2012), they have not focused specifically on adolescents or anxiety disorders. In reviews of treatment for younger children or across broad age ranges, there have been mixed findings for whether parental involvement improves outcome (Reynolds et al. 2012; Thulin et al. 2014); however, there is some indication that where parental involvement includes contingency management or transfer of control, this has a beneficial effect on child outcome at follow-up (Manassis et al. 2014). Moving forward, we need to determine whether, and how, parents of adolescents should be involved in their adolescent’s treatment for it to be most effective during this important, transitional phase of life.

This review seeks to critically evaluate the existing evidence-base, to answer the following questions:

1. In what ways have parents been involved in CBT for adolescent anxiety disorders?
2. What are the outcomes when parents are involved in CBT for adolescent anxiety disorders and is parental involvement associated with better outcomes compared to when CBT is delivered without parental involvement?

For the purpose of this review, adolescence is defined as between the ages of 11 and 18 years. This age range was selected as 11 years old is the average age for the onset of puberty (Phillips 2014), and 18 years old is typically the age that secondary or high school education and mental health services for children and young people (NHS England 2015; Public Health England 2015) come to an end, after which young people may no longer be living with their parent(s).

## Method

This systematic review follows the Preferred Reporting Items for Systematic Reviews and Meta-Analyses (PRISMA) guidelines (Moher et al. 2015).

### Search Strategy

A systematic search of relevant electronic databases (PsycINFO, Embase, CINAHL, Medline, AMED) was completed in January 2019. The search strategy used the following search terms:

(i) adolescen* OR teen* OR youth* OR young ADJ pe* OR young ADJ adult
(ii) “anxiety disorder*” OR anxi* OR phobi* OR “separation anxiety disorder” OR “generalised anxiety disorder” OR “GAD” OR “panic” OR agoraphobi* OR “social phobi*” OR “social anxi*” OR “specific phobi*” OR “specific anxi*” OR “mute” OR “mutism” OR “selective ADJ mutism”
(iii) “cognitive therap*” OR “cognitive behavi?r* therap*” OR “CBT” OR “behavio?r* therap*” OR psychotherap* OR “cognitive behavio?r* treatment” OR “cognitive behavio?r* intervention”

**Fig. 1:**
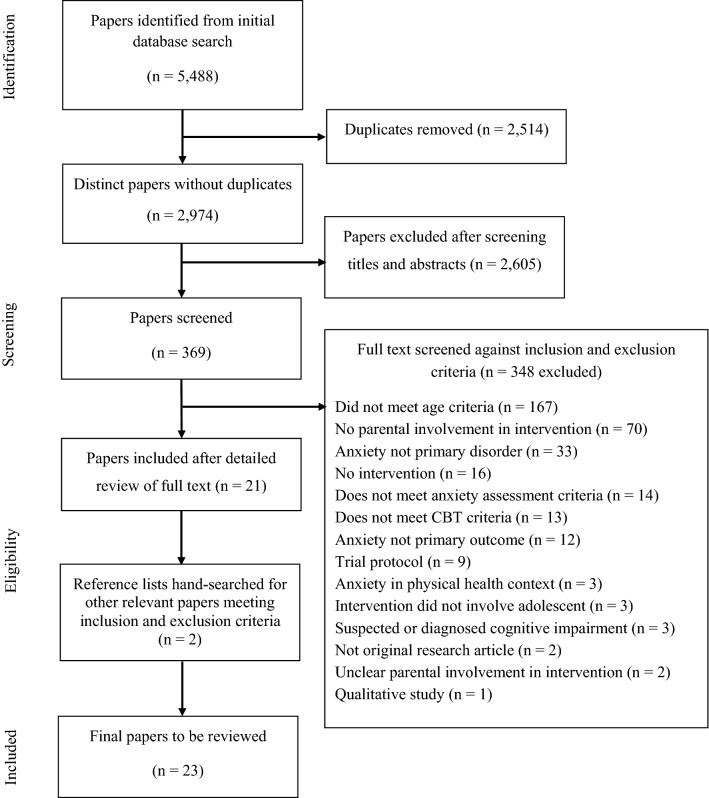
Flowchart of study selection process

In addition, Boolean operators were amended as appropriate for each database. No date ranges were specified but where possible peer-reviewed and English language limiters were used. Reference lists of selected studies and relevant reviews were hand searched to identify further papers. Any queries regarding the inclusion of a paper were discussed between the research team to agree on inclusion. A second rater (FC) reviewed the distinct papers from the search (*n* = 2974), and a Cohen’s Kappa was calculated to determine if there was agreement between the two raters (JC and FC), as to which papers should be put through to the next stage of the systematic review process. Agreement between raters was good (95.1%). The second rater also reviewed 20% (*n* = 74) of the screened papers (*n* = 369), and again, there were high levels of agreement (98.6%). A flow diagram of the search and selection process is presented in Fig. 1.

### Eligibility Criteria

Inclusion and exclusion criteria were established a priori. Studies were included if: (i) they were published in a peer-reviewed journal, (ii) they were written in English language, (iii) all adolescents within the sample were aged 11–18 years old and met diagnostic criteria for one or more anxiety disorder. Any version of the DSM may have been used to assess the presence of a clinical anxiety disorder, but adolescents had to meet the criteria of anxiety disorders as listed in the current DSM-5, thus excluding post-traumatic stress disorder and obsessive–compulsive disorder (DSM-5; American Psychiatric Association 2013). Adolescents were assessed via a (semi-) structured clinical interview that may also but did not need to include their parent(s), (iv) CBT was the treatment of the primary anxiety disorder, (v) the adolescent was included in treatment, which may have been delivered in individual face-to-face, group, family, telephone, or online/computerised formats, (vi) CBT did not have additional components from other therapeutic approaches, including pharmacotherapy, (vii) at least one biological parent was involved in treatment. At a minimum, this included their passive presence in their adolescent’s sessions. It also included their active presence in their adolescent’s sessions and/or their own parallel sessions. If there were multiple arms in the study, parents were involved in at least one arm, regardless of the type of involvement, and received the same treatment within the arm, (viii) adolescent outcomes were measured by a change in adolescent diagnostic status or anxiety symptoms pre- and post-treatment, using validated (semi-) structured interviews and/or questionnaires.

Exclusion criteria were as follows: (i) participants had an anxiety disorder(s) in the context of a physical health condition, a diagnosed or suspected neurodevelopmental disorder, learning disability, or social impairment, due to the adaptations that would need to be made to the treatment, (ii) studies that included or focused solely on foster parents, adoptive parents, carers, or guardians. The use of psychotropic medication was not an exclusion criteria.

### Data Collection

A data extraction tool was developed using guidance from the Cochrane Handbook for Systematic Reviews of Interventions (Higgins and Green 2011). The following information was extracted for each study to summarise the evidence: authors, year and location of publication, participant characteristics, recruitment, intervention, control group, additional arms if applicable, parental involvement, outcome measures, main findings, clinical implications, ethical considerations, strengths and limitations.

### Quality Appraisal

A modified version of the Downs and Black (1998) methodological quality checklist was used to critically evaluate the quality of each study according to parameters including the reporting of statistical analysis, use of valid and reliable outcome measures, and descriptions of the characteristics of the sample. The original checklist was adapted to suit the aims of this review by including an additional item: 4.a. Did the study clearly describe parental involvement? The checklist scores were categorised as excellent (27–29), good (21–26), fair (16–20), and poor (≤ 15).

### Data Synthesis

Key data and findings were extracted from the 23 papers, and the data were synthesized and organised by how parents were involved in treatment. In order to make comparisons across studies, we have reported outcomes for remission of primary anxiety disorder at post-treatment and the latest follow-up time point (where available). This can be found in Table 1. Where studies identified a primary outcome, we have also reported this data. Where no remission data are provided and multiple questionnaires were used without specifying a primary measure, the outcomes using the relevant disorder-specific measure are provided, or for treatment trials involving adolescents with mixed primary anxiety disorders, the most common general symptom measure across the studies is reported. Where reported, effect sizes in the form of Cohen’s *d* or an odds ratio (OR) are presented for the primary outcome measure of change in adolescent diagnostic status or anxiety symptomatology. Effect sizes were interpreted in line with Cohen’s (1992) conventions: an effect size of 0.2 was categorised as a small effect, 0.5 as a medium effect, and 0.8 as a large effect size, and for odds ratios, confidence intervals (CI) are provided to indicate the level of uncertainty around the measure of effect.

**Table 1:** Summary table of study characteristics, outcomes, adolescent attrition, and quality ratings

Authors, (year)		Study design	Participants: *N*; gender; age range (mean, SD), ethnicity (%)	Primary diagnosis	Intervention: name; number, duration and frequency of adolescent sessions	Control condition: name; number, duration and frequency of adolescent sessions	Diagnostic measure and primary outcome (where stated)^a^	Remission of primary diagnosis and primary outcome (where stated)^a^	Reported adolescent attrition; findings	Quality rating score
Joint parent–adolescent sessions										
Albano et al. (1995)		Case series	5; 2 female, 3 male; 13–17 (14.4); not specified	Social phobia	CBGT-A; 16, 1.5 h sessions over three months	None	ADIS-C/P	Post-treatment: not provided; 3-month follow-up: 80%; 12-month follow-up: 100%	Yes; 0%	16
Christon et al. (2012)		Case study	1; female; 15; Latina	Selective mutism and social phobia	MATCH; 60, unspecified, 21-months	None	K-SADS-PL	Selective mutism: post-treatment: 100%; no follow-up time-point. Social phobia: post-treatment: not reported; no follow-up time-point	Yes; 0%	13
Elkins et al. (2016)		RCT	54; 33 female, 21 male; 11–17 (15.29, SD 1.68); Caucasian/Non-Hispanic (86.8%) remainder unspecified	Panic disorder with (*n* = 53) or without (*n* = 1) agoraphobia	Intensive PCT-A; 8 consecutive days. Fourth and fifth days were full-day sessions (6–8 h). Treatment included 4 weekly 30-min telephone calls following the eighth day of treatment	Wait list control; 6-weeks	PDSS-C	No pre- to post/follow-up data for remission or symptom severity. PCT-A group showed significantly greater reductions in panic severity at 6-weeks than waitlist control group (*p* < 0.01) (not specified if treatment completers/ITT)	No	18
Heard et al. (1992)		Case series (multiple baseline design)	3; 3 females; 12–15 (13.33); not specified	Specific phobia	CBT; number unspecified, 1.5-h sessions over 3 months	None	FSSC-R	No pre- to post/follow-up data for remission. FSSC-R total scores decreased for all participants from pre- to post- to 3-month follow-up and were all in ‘non-clinical’ range (however, only of the 3 participants were in ‘clinical’ range pre-treatment)	Yes; 0%	15
Hoffman and Mattis (2000)		Case studies	2; 1 female, 1 male; 13 (13); not specified	Panic disorder	PCT-A; 11, 1-h, weekly	None	RCMAS	No pre- to post/follow-up data for remission. RCMAS scores moved from ‘elevated’ to within the normal range post-treatment. No follow-up data provided	Yes; 0%	13
Leyfer et al. (2018)		Pilot RCT	24; not specified; 12–17 (14.5, SD 1.77); Non-Hispanic White (95.8%), African American (4.2%)	Panic disorder with agoraphobia	Intensive PCT-A + DCS; 8 consecutive days of 2–6-h treatment each day	Intensive PCT-A + placebo; 8 consecutive days of 2–6-h treatment each day	ADIS-C/P	Post-treatment remission (treatment completers): 66.7% CBT + DCS group, 90% CBT + placebo group (differences not significant, *p* = .32). 3-month follow-up remission (treatment completers): 83.3% CBT + DCS group, 90% CBT + placebo group (differences not significant, *p* = 1.0)	Yes; 8.33% (CBT + DCS: 16.67%, CBT + placebo: 0%) dropped out during treatment	25
Ollendick (1995)		Case series (multiple baseline design)	4; 3 female, 1 male; 13–17 (15); Caucasian (100%)	Panic disorder with agoraphobia	CBT for panic with agoraphobia; 10–12 (+ 2 booster sessions in following month), unspecified, weekly	None	ADIS-C/P	Post-treatment remission: 100%; 6-month follow-up remission: 100%	Yes; 0%	16
Pincus et al. (2010)		RCT	26; 19 females, 6 males; 14–17 (15.75, SD 1.10); Caucasian (100%)	Panic disorder with agoraphobia	PCT-A; 11, 50-min weekly sessions, over a 12-week period (additional week between session 11 and 12)	Self-monitoring group; 20–30-min sessions, biweekly, over 8 weeks, to monitor panic and mood symptoms	ADIS-C/P (CSR)	Remission data not provided. PCT-A group (ITT) showed significantly greater reductions in CSR scores than control group (*p* < .01, *d* = 1.09). PCT-A (combined sample^b^) CSR effect sizes pre- to post-treatment were large: *d* = 2.17. At follow-up CSR scores continued to decrease from post-treatment to 3-month follow-up (*p* < .01) and ‘did not change’ from 3- to 6-month follow-up. (not specified if treatment completers/ITT)	Yes; 12% (PCT-A: 12% dropped out of treatment, control: 0%)	20
Separate parent sessions										
Anderson et al. (1998)		Case study	1; male; 13; not specified	Social phobia and adjustment disorder with anxiety	CBT with parent and school involvement; 7, 3 weeks, unspecified	None	ADIS-C/P	Post-treatment: 100% remission. 5-month follow-up: 100% remission	Yes; 0%	11
Baer and Garland (2005)		RCT	12; 7 females, 5 males; 13–18 (15.5); not specified	Social phobia	Modification of SET-C; 12 1.5-h, weekly group sessions	Waitlist: details not specified	ADIS-C	Post-treatment remission (treatment completers): treatment group: 64%, control group 0%. Treatment group improved significantly compared to the waitlist control (*p* = 0.03; *d* = 1.63). No follow-up data provided	Yes; 8.33% in SET-C dropped out of treatment	18
Legerstee et al. (2008)		RCT	51; 29 females, 22 males; 12–16 (13.9, SD 1.1; not specified	SAD, GAD, social phobia, specific phobia, panic disorder, agoraphobia	Individual CBT using the Dutch translation of FRIENDS program; 10 unspecified, weekly	(adolescents in the trial only received individual CBT, whereas children, who were analysed separately, were randomised to either group or individual CBT)	ADIS-C/P	Post-treatment remission (ITT): 64%. Maternal (but not paternal) lifetime anxiety disorders were a significant predictor of remission (*p* = 0.02, OR 6.36, 95% CI 1.30–31.11). No follow-up data were provided	No	20
Masia-Warner et al. (2005)		RCT	42; 26 females, 9 males; 14–17 (14.8, SD 0.81); Caucasian (82.9%), African American (8.6%), Asian American (2.9%), Latin American (2.9%), Other (2.9%)	Social anxiety disorder	SASS; 12 40-min weekly group school sessions, 2 15-min individual meetings, 4 90-min social events, 2 monthly group booster sessions, over 3 months	Waitlist: details not specified	ADIS-C/P LSAS-CA	Post-treatment remission (treatment completers): SASS group 67%, control group 6%. SASS led to significantly greater CSR reductions (*p* < 0.0001, *d* = 2.4) than the control group. 9-month follow-up remission (treatment completers): SASS group 72% (no control comparison)	Yes; 16.67% in SASS dropped out of treatment	19
Masia-Warner et al. (2007)		RCT	36; 30 females, 6 males; 14–16 (15.1, SD 0.6); Caucasian (72.2%), African-American (5.6%), Hispanic (16.7%), Other (5.6%)	Social anxiety disorder	SASS; 12 40-min group sessions, 2 15-min individual sessions, 4 90-min social events, 2 booster sessions	Educational-Supportive Group Function (ESGF); format and therapist contact identical to SASS	ADIS-C/P (CSR) CGI	Post-treatment remission (treatment completers): SASS group 58.8%, control group 0%. SASS led to significantly more adolescents in remission (*p* < .001) than the control group. 6-month follow-up remission (treatment completers): SASS 73.3%, control 6.7%; difference between groups was significant (*p* < .01)	Yes; 11.11% (SASS group: 10.52%, ESGF: 11.76%) dropped out of treatment	22
Masia-Warner et al. (2016)		RCT	138; 94 female, 44 male; 14–17 (15.42, SD 0.81); White (72%)	Social anxiety disorder	C-SASS and P-SASS; 12 group sessions, 2 15-min individual sessions, 4 90-min social events, two group booster sessions	Skills for Life (SFL); non-specific counselling program, details unspecified	ADIS-C/P (CSR)	Post-treatment remission (ITT): C-SASS 20.9%, P-SASS 30.8%, SFL 7.9%. 5-month follow-up remission (ITT): C- SASS: 39.5%, P-SASS 33.3%, SFL 13.2%. No significant differences between C-SASS and P-CASS on any outcomes. SASS had significantly lower CSR scores than controls at post-treatment (C-SASS *d* = 0.69, P-SASS *d* = 0.67) and 5-month follow-up (C-SASS *d* = 0.93, P-SASS *d* = 0.83)	Yes; 13.04% (C-SASS: 6.52%, P-SASS: 17.02%, SFL: 11.63%) dropped out of treatment	21
Nordh et al. (2017)		Case series	30; 25 females, 5 males; 13–17 (15, SD 1.22); not specified	Social anxiety disorder	Internet-delivered CBT; 12 weeks, 9 remote therapist-guided internet-delivered sessions and 3 2-h group exposure sessions on weeks 4, 6, 10	None	MINI KID ADIS-C Social anxiety disorder section, CGI-S (specified as primary)	Post-treatment remission: 47% (*d* = 1.17). 6-month follow-up: 57% (*d* = 0.22). (86.67% of sample assessed, not specified if treatment completers). CGI-S (ITT) decreased pre- to post-, *p* < .001, *d* = 1.17, and from post- to 6-month follow-up, *p* < .05, *d* = 0.22	Yes; 36.67% completed 7–9 internet sessions and 2/3 attended 2–3 group sessions	22
Spence et al. (2008)		Case study	1; female; 17; Caucasian	Social phobia	BRAVE-for teenagers ONLINE; 10, 1-h, weekly, with 2 booster sessions	None	ADIS-C/P CGAS	Post-treatment remission: 100%. Follow-up data not reported	Yes; 0%	13
Spence et al. (2011)		RCT	115; 68 female, 47 male; 12–18 (13.98, SD 1.63); not specified	GAD (48%), social phobia (35%), SAD (13%), specific phobia (4%)	BRAVE-for teenagers ONLINE (NET), 10, 1-h, weekly, 2 booster sessions at 1- and 3-months post-treatment BRAVE-CLINIC (CLIN), 10, face-to-face, 1-h, weekly, 2 booster sessions at 1- and 3-months post-treatment	Waitlist: 12 weeks with no contact	ADIS-C/P CGAS	Remission 12-weeks post-treatment (ITT): NET: 34.1% CLIN 29.5%: control: 3.7%. 12-month follow-up remission (ITT): NET 68.2%, CLIN 68.2% (no control data). No significant differences at 12-month follow-up between NET and CLIN (*p* = 1.00)	Yes; 12-month follow-up 43% NET and 21% CLIN adolescents did not complete all 10 sessions. This difference was statistically significant (*p* = .02)	22
Waite et al. (2019)		RCT	60; 39 female, 21 male; 13–18 (14.7, SD 1.34); White British (91.7%), remainder of sample unspecified	Social anxiety disorder, GAD, specific phobia, SAD, panic with or without agoraphobia, agoraphobia	BRAVE-for teenagers ONLINE; 10, 1-h, weekly Two arms: Adolescent and parent (ADOL + PARENT) Adolescent only (ADOL-ONLY)	Waitlist: 10 weeks with no contact	ADIS-C/P (remission specified as primary)	*CBT versus waitlist:* Post-treatment remission (ITT): intervention 40.0%, control 33.3% (p = 0.59. Difference not statistically significant (*p* = 0.12, OR 1.33, 95% CI 0.46–3.82). 6-month follow-up remission (ITT): post-CBT 51.7% (no control data). Significant improvements from post-CBT to 6-month follow-up (p = .04, OR = 13.72, 95% CI 0.77–12.60) *Parent involvement:* Post-CBT remission (ITT): ADOL + PAR 33.3%, ADOL-ONLY 40.0%. Difference not significant (p = 0.59, OR 0.75, 95% CI 0.26–2.15). 6-month follow-up remission (ITT): ADOL + PAR 53.3%, ADOL-ONLY 50.0%. Difference not significant (*p* = 0.80, OR 1.14, 95% CI 0.42–3.15). (all analyses ITT)	Yes; 20.7% did not complete all 10 sessions 21.43% ADOL + PAR and 13.33% ADOL-ONLY did not complete post-CBT assessment	25
Separate parent sessions and joint parent–adolescent sessions										
Kendall and Barmish (2007)		Case study	1; male; 13; Caucasian	Social phobia	Coping Cat; 14 unspecified duration, weekly	None	ADIS-C/P	Post-treatment remission: 100%. No follow-up data reported	Yes; 0%	10
Siqueland et al. (2005)		Phase I Case series	8; 4 females, 4 males; 14–17 (15.5); Caucasian (87.5%), Hispanic (12.5%)	GAD (75%), social phobia (25%)	CBT-ABFT; 16; unspecified duration and frequency	None	BAI	Post-treatment remission data not reported. 88% of BAI scores in ‘non-clinical’ range (≤ 18)	Yes; 0%	18
		Phase II RCT	11; 3 females, 8 males; 12–17 (14.9, SD 1.8); Caucasian (90.9%), African American (9.1%)	GAD (90.9%), SAD (9.1%)	CBT-ABFT; 16; unspecified duration and frequency	CBT modified for adolescents; 16; unspecified duration and frequency	ADIS-C/P BAI CRPBI	Post-treatment remission (all participants): CBT-ABFT 40%, CBT 67%. 6-month follow-up remission: CBT-ABFT 80%, CBT 100%	Yes; CBT-ABFT 9.09%, CBT 0% did not complete 12/16 sessions (‘adequate dose’)	17
Workbook										
Stjerneklar et al. (2018)		Case series (multiple baseline design)	6; 3 females, 3 males; 13–17 (15); not specified	GAD, social phobia, specific phobia	Internet-based Chilled Out; 12-weeks to complete 8 online modules, 30-min each	None	ADIS-C/P	Post-treatment remission (all participants) 33.33%. 3-month follow-up data not reported	Yes; 16.67% dropped out of treatment	16
Wuthrich et al. (2012)		RCT	24; 16 females, 8 males; 14–17 (15.17, SD 1.11); Australian (77.3%), Asian / Asian Australian (4.5%), European/European Australian (13.6%), Other (4.5%)	GAD, social phobia, SAD, specific phobia, anxiety disorder not otherwise specified	Cool Teens; 8 therapy modules of 30-min, duration of treatment not specified	Waitlist: 12 weeks no contact	ADIS-C/P	Post-treatment remission (treatment completers): Cool Teens 41%; control 0%. 3-month follow-up: Cool Teens 26% (no control group data)	Yes; 8.33% dropped out of treatment	21
Format not specified										
Leigh and Clark (2016)		Case series	5; 4 females, 1 male; 11–17 (14.8); not specified	Social anxiety disorder	CT-SAD; 14, 1.5 h, with follow-up at 1, 2, 3 months post-treatment	None	ADIS-C/P LSAS (specified as primary)	Post-treatment: 100% remission; no follow-up remission data. LSAS showed symptom severity improved from pre- to post and post- to 2-3-month follow-up	Yes; 0%	16

ABFT = Attachment-based Family Therapy, ADIS-C/P = Anxiety Disorders Interview Schedule for Children, Child and Parent Versions, BAI = Beck Anxiety Inventory, CASI = Childhood Anxiety Sensitivity Index, CBCL = Child Behaviour Checklist, CBGT-A = Cognitive-Behavioural Group Treatment for Adolescents, CBT = Cognitive Behavioural Therapy, CDI = Children’s Depression Inventory, CGAS = Children’s Global Assessment Scale, CGI-S = Clinical Global Impression-Severity, CI = Confidence Interval, CIDI = Composite International Diagnostic Interview, CRPBI = Children’s Report of Parenting Behaviour Inventory, C-SASS = SASS delivered by school counsellors, CSR = Clinical Severity Rating, CT-SAD = Cognitive Therapy for Social Anxiety Disorder, DAS = Dyadic Adjustment Scale, DCS = D-cycloserine, FSSC-R = Fear Survey Schedule for Children – Revised, GAD = Generalised Anxiety Disorder, HAM-A = Hamilton Anxiety Rating Scale, ITT = intention to treat analysis, K-SADS-PL = Kiddie Schedule for Affective Disorders and Schizophrenia-Present and Lifetime Version, LSAS-CA = Liebowitz Social Anxiety Scale for Children and Adolescents, MASC = The Multidimensional Anxiety Scale for Children, MATCH = Modular Approach to Therapy for Children with Anxiety, Depression, Trauma, or Conduct Problems, MINI KID = Mini International Neuropsychiatric Interview for Children and Adolescents, OR = Odds Ratio, PCT-A = Panic Control Treatment for Adolescents, PDSS-C = Panic Disorder Severity Scale for Children, P-SASS = SASS delivered by psychologists, RCANXIETY DISORDERS-C/P = Revised Child Anxiety and Depression Scale-Child/Parent form, RCMAS = Revised Children’s Manifest Anxiety Scale, RCT = Randomised-Controlled Trial, SAD = Separation Anxiety Disorder, SASS = Skills for Academic and Social Success, SD = Standard Deviation, SET-C = Social Effectiveness Training for Children and Adolescents, SPWSS = Social Phobia Weekly Summary Scale. Quality ratings: ≤ 15 = poor, 16–20 = fair, 21–26 = good, 27—29 = excellent

^a^If there was no diagnostic measure or stated primary outcome measure, the measure most closely related to the target disorder was selected

^b^Combined sample comprised of adolescents who completed PCT-A immediately and those who completed PCT-A after the self-monitoring control condition

## Results

### Study Characteristics

The 23 papers were published between 1992 and 2019 and contained 24 studies (Siqueland et al.’s (2005) paper contained two studies). A total of 18 research groups conducted the 24 studies. Table 1 provides detailed information on study characteristics. Twelve of the papers report on studies conducted in the USA, five in Australia, two in the United Kingdom, one in Canada, one in Denmark, one in the Netherlands, and one in Sweden. Study design included five case studies, seven case series (three using multiple baseline design), and 12 randomised-controlled trials.

Within the 24 studies, sample sizes ranged from 1 to 138, with a mean sample size of 27.74. The mean age of participants ranged from 13.33 to 15.75 years. Eighteen studies included adolescents of both genders. Eighteen studies were based within outpatient clinics, and six studies did not report the setting. Eleven studies included participants who were on psychotropic medication, and all ensured that participants were on a stable dosage prior to starting CBT. Thirteen studies investigated individual face-to-face CBT for adolescents, ranging from 7 to 60 sessions (mean number of sessions = 15.36). The duration of treatment ranged from 3 weeks to 21 months. Six studies investigated online CBT, with programs comprising 8 to 12 sessions, across 10 to 12 weeks. Five studies investigated group CBT, ranging from 12–16 sessions, with the duration of sessions ranging from 40 to 90 min. Over two thirds of the studies (*k* = 16) did not specify parent gender; where gender was specified (typically in case studies/series), four studies included the adolescent’s mother and four studies included mothers and fathers. No study reported parent characteristics of age, socio-economic status or ethnicity.

Studies reported on outcome measures that related to the adolescent’s anxiety symptoms and/or diagnostic status. Only three studies identified a primary outcome measure (each different).

### Quality Appraisal

The Downs and Black (1998) checklist was used to structure and guide the quality appraisal. Online Resource 1 provides full details of the quality appraisal for each study. The quality of studies was rated, and studies were categorised as: ≤ 15 = poor, 16–20 = fair, 21–26 = good, 27–29 = excellent. Total scores across the 24 studies ranged from 10 to 25. Six studies received a quality rating of poor, eleven received a quality rating of fair, and seven received a quality rating of good. No studies received a quality rating of excellent.

All of the studies, except Legerstee et al. (2008), clearly described the interventions. Sixteen papers clearly described parental involvement. However, seven papers lacked detailed information regarding parental involvement, including when in the treatment process parents were involved, in what way parents were involved and what parental involvement comprised of (Baer and Garland 2005; Legerstee et al. 2008; Leigh and Clark 2016; Masia-Warner et al. 2005; Masia-Warner et al. 2007; Ollendick 1995; Stjerneklar et al. 2018). All the studies used valid and reliable primary outcome measures. The impact of bias in the results was compromised in many studies due to a lack of accounting for confounders and dropouts. Only Pincus et al. (2010) and Waite et al. (2019) used multiple imputation methods to account for missing data. Overall, studies failed to provide sufficient detail to determine how representative participants were of the entire population, including poor reporting of ethnicity, as well as randomisation and blinding procedures. While seven of the RCTs demonstrated sufficient power (Leyfer et al. 2018; Masia-Warner et al. 2007, 2016; Pincus et al. 2010; Spence et al. 2011; Waite et al. 2019; Wuthrich et al. 2012), it is unclear whether power calculations were conducted in the remaining five RCTs. Across the studies, sample sizes were generally small; thus, it is possible that many studies were underpowered.

### Research Question 1: In What Ways Have Parents Been Involved in CBT for Adolescent Anxiety Disorders?

Table 2 summarises how parents were involved in the studies, including the number and duration of parent sessions, the treatment components that parents were involved in, as well as parent satisfaction with treatment.

**Table 2:** Nature of parental involvement, treatment components involving parents, key findings, and parent satisfaction with treatment

Authors (year)	Parent(s) relationship to child	Nature of parental involvement	Number (duration) of each parent session	Treatment components involving parents								Parent satisfaction with treatment
				Psycho-education	Relaxation	Problem solving	Cognitive restructuring	Contingency Management	Supporting graded exposure	Addressing parental beliefs and behaviours	Relapse prevention	
Joint parent–adolescent sessions												
Albano et al. (1995)	Not specified	Joined adolescent sessions 1, 2, 8, 15	4 (90-min)	X	–	–	–	–	X	X	–	Not reported
Christon et al. (2012)	Mother	Majority of sessions included 10- to 15-min parent component or review of treatment	Not specified	–	–	–	–	X	X	X	–	Not reported
Elkins et al. (2016)	Not specified	Unclear involvement in sessions	Not specified	–	–	–	–	–	X	–	–	Not reported
Heard et al. (1992)	Mothers and fathers	Joined adolescent sessions	Weekly (90-min) for 3-months	–	–	–	–	X	X	–	–	Not reported
Hoffman and Mattis (2000)	Mother/not specified	Joined the end of adolescent sessions 1, 4, 7, 11	4 (60-min)	X	–	–	X	–	X	–	–	Not reported
Leyfer et al. (2018)	Not specified	Parent component at the end of each adolescent session. Parent involvement was identical in both arms	6 (30-min)	X	–	–	X	X	X	–	X	Not reported
Ollendick (1995)	Mother	Joined adolescent sessions	Not specified	–	–	–	–	X	X	–	–	Not reported
Pincus et al. (2010)	Not specified	Joined the end of adolescent sessions 1, 4, 7, 11	5 (10-min)	X	–	–	–	X	X	X	–	Reported that parents felt the best part of treatment was learning a common language to use with adolescent and learning how to best help their adolescent while experiencing a panic attack (measures and participant numbers not specified)
Separate parent sessions												
Anderson et al. (1998)	Not specified	Separate parent sessions	7 (not specified)	X	–	–	–	X	–	–	–	Not reported
Baer and Garland (2005)	Not specified	Separate parent group session	1 (not specified)	X	–	–	–	–	–	–	–	Not reported
Legerstee et al. (2008)	Not specified	Separate parent sessions	4 (90-min)	–	–	–	–	–	–	–	–	Not reported
Masia-Warner et al. (2005)	Not specified	Parent group sessions	2 (45-min)	X	–	–	–	X	–	–	–	Not reported
Masia-Warner et al. (2007)	Not specified	Parent group sessions	2 (45-min)	X	–	–	–	X	–	–	–	4 questions assessing views of therapist skill, knowledge, overall satisfaction, and likelihood of recommending SASS. Reported that parents of adolescents in SASS group had significantly higher ratings than the attention control group (p < .05) but satisfaction was not related to parent ratings of improvement (participant numbers not specified)
Masia-Warner et al. (2016)	Not specified	Parent group sessions	2 (45-min)	X	–	–	–	X	–	–	–	Not reported
Nordh et al. (2017)	Not specified	Separate internet-delivered parent sessions	5 (not specified)	X	-	X	–	X	X	X	X	Not reported
Spence et al. (2008)	Mother	Separate internet-delivered parent sessions	5 (not specified)	X	X	X	X	X	X	–	–	Mother completed 8-item questionnaire. Reported ‘high levels’ of satisfaction and that the program had taught skills to manage anxiety and cope better with anxiety-provoking situations
Spence et al. (2011)	Not specified	Separate internet-delivered or face-to-face parent sessions dependent on treatment arm	5 (60-min) 2 booster sessions (not specified)	X	X	X	X	X	X	–	–	88.64% of parents completed an adapted questionnaire. Reported moderate to high satisfaction, although parents in face-to-face condition reported ‘slightly higher’ satisfaction
Waite et al. (2019)	Not specified	Separate internet-delivered parent sessions	5 (not specified)	X	X	X	X	X	X	–	–	71.7% (97.7% treatment completers). Reported that 95.3% of parents who had completed parent sessions and 81.9% of parents who had not completed parent sessions were ‘moderately’ to ‘extremely’ satisfied with their adolescent’s treatment
Separate parent sessions and joint parent–adolescent sessions												
Kendall and Barmish (2007)	Mother and father	Separate parent sessions and attended adolescent sessions	2 (60-min) 5 (60-min)	X	–	–	–	–	X	X	–	Not reported
Siqueland et al. (2005)^a^	Mothers and fathers	Separate parent sessions in CBT-only arm	2 (not specified)	–	–	–	–	–	X	–	–	Not reported
		Separate parent sessions and parent–adolescent sessions in CBT-ABFT arm	2 (not specified)	–	–	–	–	X	X	X	–	In informal exit interviews, parents in CBT-ABFT arm were reported to find the family work to be the ‘most important or satisfying’ treatment component. ‘Some’ parents in CBT alone arm ‘expressed disappointment’ in the limited parental involvement in treatment (participant numbers not specified)
Workbook												
Stjerneklar et al. (2018)	Not specified	Separate parent workbook, phone calls with therapist and individualised involvement in adolescent sessions	Not specified	X	–	–	X	–	X	–	–	83.33% of parents completed CHI-ESQ; parents were reported to be ‘generally satisfied’ with the intervention, although 1/3 of parents would have liked a face-to-face meeting pre-treatment with the therapist/other families
Wuthrich et al. (2012)	Mothers	Separate parent workbook and individualised involvement in adolescent sessions	4 (not specified)	X	–	–	–	–	X	–	–	Not reported
Format not specified												
Leigh and Clark (2016)	Not specified	Individualised	Not specified	X	–	–	–	X	–	X	X	100% of parents completed CHI-ESQ. Reported ‘high level’ of satisfaction as all endorsed all items as ‘partly’ or ‘certainly’ true

ABFT = Attachment-based Family Therapy, CBGT-A = Cognitive-Behavioural Group Treatment for Adolescents, CBT = Cognitive Behavioural Therapy, CHI-ESQ = The Experience of Service Questionnaire (Attitude-Stirling, 2002), CT-SAD = Cognitive Therapy for Social Anxiety Disorder, DCS = D-Cycloserine, MATCH = Modular Approach to Therapy for Children with Anxiety, Depression, Trauma, or Conduct Problems, PCT-A = Panic Control Treatment for Adolescents, SASS = Skills for Academic and Social Success, SET-C = Social Effectiveness Training for Children and Adolescents

^a^The paper by Siqueland et al. (2005) involved two studies—parents in each study received the same treatment

#### Format of Parental Involvement

Half the studies (*k* = 12) provided separate sessions for parents. Four were delivered online, four in a parent group, three through individual face-to-face sessions, and in the remaining study it was not specified whether this was individually or within a group (Legerstee et al. 2008). Of the online studies, three used the BRAVE Program for Teenagers, in which parents were offered five sessions and two post-treatment booster sessions alongside their adolescent’s online treatment (Spence et al. 2008, 2011; Waite et al. 2019) and one used ‘BIP SOFT’, involving five parent modules (Nordh et al. 2017). Spence et al. (2011) also included an individual face-to-face CBT treatment arm, in which parental involvement mirrored that of the online BRAVE treatment, with parents independently completing a workbook rather than an online programme. Three studies involving groups for parents offered sessions as part of the Skills for Academic and Social Success (SASS) intervention (Masia-Warner et al. 2005; Masia-Warner et al. 2007, 2016) and comprised of two 45-min sessions. A further study involved one parent group session to provide information and answer questions about the adolescent’s treatment (Baer and Garland 2005).

The three studies involving individual face-to-face sessions with parents consisted of two sessions (Kendall and Barmish 2007), seven sessions (Anderson et al. 1998) and up to nine sessions depending on the individual case (Siqueland et al. 2005).

In over a third of the studies (*k* = 10), parents joined the adolescent’s sessions. Four studies involved the parents in all the adolescent’s session, either for the whole session (Heard et al. 1992; Ollendick 1995) or at the beginning/end of the session (Christon et al. 2012; Leyfer et al. 2018). Three studies involved parents in four key sessions, either for the whole session (Albano et al. 1995) or at the end (Hoffman and Mattis 2000; Pincus et al. 2010). Parents also attended sessions with their adolescents in two of the studies that provided separate parent sessions (Kendall and Barmish 2007; Siqueland et al. 2005). In Kendall and Barmish (2007), the parents appeared to attend some of two of the adolescent’s sessions and in Siqueland et al. (2005), there was one joint parent–adolescent session at the beginning and then up to a further eight joint sessions depending on the case. The final study did not report how many of the adolescent sessions parents joined or whether they were present for part or the whole of the adolescent’s session (Elkins et al. 2016).

One study offered individualised sessions that involved parents if problematic parental beliefs and behaviours were identified (Leigh and Clark 2016). However, it was not stated whether this was within the adolescent’s session or delivered as separate sessions to parents.

Less than 10% of studies (*k* = 2) offered parents a hard-copy workbook to engage with throughout their adolescent’s computerised/internet-delivered treatment (Stjerneklar et al. 2018; Wuthrich et al. 2012). These two studies reported that parents were encouraged to support their adolescent in completing their sessions, but the extent to which they did this was determined by the adolescent. Parents could also contact their adolescent’s therapist, which was flexibly arranged in the former study and allocated at specific sessions in the latter study.

#### Content of Parental Involvement

The aim of parental involvement across the studies was to support their adolescent’s treatment. Parental involvement primarily consisted of developing both an understanding of the core components of CBT (i.e. psychoeducation, relaxation training, cognitive restructuring, graded exposure, and problem solving), skills in managing the adolescent’s difficulties (i.e. contingency management), and addressing the parent’s own (potentially unhelpful) beliefs so that they did not interfere with the adolescent’s progress in treatment. The following sections are presented in order of how frequently the studies reported the content being included in parent sessions.

#### Psychoeducation

Around three quarters of the studies (*k* = 17) reported that parents were provided with psychoeducation. This comprised of educating parents about the nature and aetiology of anxiety disorders, as well as orienting them to CBT, including providing a rationale for its use and helping manage their expectations of treatment. The predominant aim of providing parents with psychoeducation was to develop their understanding of their adolescent’s difficulties and to help them support their adolescent through treatment.

#### Supporting Graded Exposure

Around two thirds of the studies (*k* = 16) reported that parents were involved in supporting graded exposure. This included discussion of the distinct roles of parents and adolescents within exposure tasks, as well as how parents could support exposure tasks within and outside of sessions. Parents were viewed as ‘coaches’, supporting their adolescent to engage in graded exposure outside of treatment sessions throughout the course of treatment. Two studies explicitly stated that parents were also involved in the development of their adolescent’s exposure hierarchy (Christon et al. 2012; Kendall and Barmish 2007).

#### Contingency Management

Just under two thirds of the studies (*k* = 15) reported that parents were taught contingency management techniques, with similar content across the studies. Parents were taught techniques to help manage their adolescent’s anxiety disorder, learning to use praise statements and to stop reinforcing their adolescent’s avoidance. In this way, parents were supported to be able to help their adolescents effectively deal with anxiety-provoking situations, reducing their adolescent’s use of safety behaviours and their own use of reassurance, thus reducing family accommodation of the difficulties.

#### Addressing Parental Beliefs and Behaviours

Just under a third of studies (*k* = 7) included parents in discussions regarding how their own beliefs and behaviours may have an impact on their adolescent’s difficulties. In the CBT plus attachment-based family therapy (ABFT) arm of the Siqueland et al. (2005) studies, parents engaged in joint sessions with their adolescent to directly address family dynamics in the context of their adolescent’s anxiety. This largely involved discussions regarding parents’ anxieties and fears when facilitating their adolescent’s autonomy and challenging anxiety through the process of therapy. Three studies also offered parents separate sessions (where relevant) to explore their beliefs about their adolescent’s anxiety and the impact of these beliefs (Leigh and Clark, 2016), attempting to change parental attitudes (Anderson et al. 1998) and offer them the opportunity to understand their own reactions to their adolescent (Nordh et al. 2017). Albano et al. (1995) included discussion of communication in the parent–adolescent dyadic relationship, as well as parents’ concerns, expectations, and goals for treatment. A further study included discussion of the importance of parents and adolescents spending time together (Christon et al. 2012). Kendall and Barmish (2007) also incorporated discussion of the transference of control from therapist to parent and subsequently adolescent, seeking to facilitate the maintenance of change.

#### Cognitive Restructuring

Around a quarter of studies (*k* = 6) reported that they involved parents in cognitive restructuring (Spence et al. 2008, 2011; Waite et al. 2019; Hoffman and Mattis 2000; Leyfer et al. 2018; Stjerneklar et al. 2018). Hoffman and Mattis (2000) described parents joining the end of the session to discuss the material covered in the adolescent’s session that related to automatic thoughts, probability overestimations, and how to counter them through ‘being a detective’. In Spence et al. (2008; 2011) and Waite et al. (2019), this involved the parent learning about coping self-talk and cognitive restructuring within their sessions.

#### Problem Solving

17.4% of studies (*k* = 4), all delivering parent sessions online, reported that parents were involved in problem solving (Nordh et al. 2017; Spence et al. 2008, 2011; Waite et al. 2019). However, no detail was provided regarding the content of problem solving.

#### Relaxation Training

13.0% of studies (*k* = 3), all involving the online BRAVE program, reported that they delivered relaxation to parents (Spence et al. 2008, 2011; Waite et al. 2019). Parents and adolescents were provided with a relaxation CD to complement their online sessions.

#### Relapse Prevention

13.0% of studies (*k* = 3) stated that parents were included in relapse prevention planning (Leyfer et al. 2018; Leigh and Clark, 2016; Nordh et al. 2017). In Leigh and Clark (2016), parents were invited to join their adolescent’s final session, and the adolescent was encouraged to share their relapse prevention plan with their parents, alongside discussion of the parents’ role in supporting implementation of the plan. In Nordh et al. (2017), parents completed an online module to help them ‘prepare relapse prevention’. In the study by Leyfer et al. (2018), parents were also involved in relapse prevention as part of the treatment. However, the description of their exact involvement in this component of treatment was unclear.

### Research Question 2: What are the Outcomes When Parents are Involved in CBT for Adolescent Anxiety Disorders and is Parental Involvement Associated with Better Outcomes Compared to When CBT is Delivered Without Parental Involvement?

Table 1 summarises the study characteristics and findings (attrition as well as clinical outcomes) from each of the 24 studies. Twelve of the identified studies reported on case studies or series (including multiple baseline designs). All of these case studies/series (including parents for different durations, in different formats, and with different content) reported reductions in adolescent anxiety symptoms and disorder from pre- to post-treatment. Remission rates of primary diagnosis ranged from 33.33 to 100% (with three quarters of the studies reporting 100% of adolescents in remission (six of the eight studies). Where studies only reported outcomes on symptom measures (*k* = 4), 88–100% participants in each study were in the ‘non-clinical’ range at the end of treatment. Where studies included longer-term follow-ups (*k* = 6), there was evidence that reductions in anxiety were maintained for up to 12 months (e.g. Albano et al. 1995).

RCTs showed much greater variability of remission rates, with studies finding between 20.9% and 90% of the sample were free of their primary diagnoses post-treatment. Nevertheless, eleven of the twelve studies that compared the treatment to a waitlist or no treatment control found significant benefits of treatment. Eight of the studies included longer-term follow-ups, and all but one study (Wuthrich et al. 2012) showed a greater number of adolescents in remission at follow-up than at post-treatment. Three studies included an active control that included similar format and extent of parent involvement, so these studies are not able to provide any information about outcomes on the basis of parent involvement.

Across the studies, there was no clear pattern of effect according to the format or content of parent involvement. For example, studies involving separate parent sessions showed remission rates ranging from 21 to 100%, and variability in outcomes even between studies evaluating the same program (e.g. remission rates for the SASS program ranged from 21 to 67%, Masia-Warner et al. 2005, 2007, 2016). Studies that reported teaching parents how to support the adolescent in doing graded exposure had outcomes ranging from 33 to 100% and those that did not report this being included in sessions had outcomes ranging from 21 to 100%. Similarly, when contingency management was reported to be included, outcomes were in the range of 21–100% and where it was not, outcomes were in the range of 33–100%. Where studies that were rated as low quality were removed from the analysis, the general pattern of results was maintained, i.e. that there was evidence that treatments were broadly effective regardless of the extent, format or content of parental involvement.

In terms of treatment acceptability, few studies measured this in a systematic way. Where parents were asked for feedback (typically those completing treatment), this was universally positive, although notably in one study of CBT plus ABFT (Siqueland et al. 2005), parents who were involved in CBT only (which involved them supporting young people with graded exposure) reported disappointment at the lack of parental involvement, and those who received ABFT rated this component of treatment as the most important or satisfying aspect of treatment. Where reported, attrition was generally low (between 0 and 21%).

Finally, one study compared CBT for adolescents with anxiety disorders with and without parent involvement in treatment sessions (Waite et al. 2019). Parent sessions did not lead to significant improvements post-treatment (*p* = 0.59, OR 0.75, 95% CI 0.26–2.15) or at 6-month follow-up (*p* = 0.80, OR 1.14, 95% CI 0.26–2.15). Post-treatment, parents completed questions about their involvement in their adolescent’s treatment and the majority of parents had provided some support to their adolescent in completing the program (regardless of whether they were offered specific parent sessions). Notably, twice as many adolescents dropped out of treatment in the group that had parental involvement compared to the group with adolescent-only sessions (21.43% versus 13.33%); however, a greater number of parents who had completed parent sessions were satisfied with the overall treatment than those who had not completed parent sessions (95% vs. 82%) and there were lower rates of onward referral for further input for adolescents whose parents had completed sessions.

## Discussion

This is the first systematic review to focus on parental involvement in CBT for anxiety disorders *for adolescents specifically*. We identified 24 studies and found that parents were involved in their adolescent’s treatment for a wide range of different durations and in different formats. Content varied but was most typically aimed at the parent developing an understanding of core CBT components, e.g. psychoeducation and supporting graded exposure, and skills to help them manage their adolescent’s anxiety and avoidance. Almost all the studies showed significant benefits of treatment in both the short-term and at longer-term follow-up, relatively low attrition and high levels of parent satisfaction, and no clear pattern of effect according to the format of parent involvement. Only one of the studies (Waite et al. 2019) allowed us to examine outcomes on the basis of parent involvement and found that providing additional (internet-delivered) parent sessions did not lead to significantly improved outcomes either post-treatment or at follow-up.

The methodologies of the studies meant that it was not possible to evaluate the specific contribution of parental involvement to treatment outcomes for adolescents. Only half the studies were RCTs and all but one compared CBT (that included parents), with either a waitlist or active control condition and did not involve a comparator arm comprising CBT without parental involvement. This is in contrast to studies with younger children or children across broad age ranges, where a much large number of studies have directly compared parent-involved with child-only treatments; for example, Thulin et al. (2014) meta-analysis identified 16 studies that made these direct comparisons. However, even with this larger body of evidence, meta-analyses have produced mixed finding for whether parental involvement improves outcomes (e.g. Reynolds et al. 2012; Thulin et al. 2014; Kreuze et al. 2018).

The wide variation in how parents have been involved makes it hard to draw conclusions about whether particular types of involvement are beneficial for adolescents. As far as we were able to tell, the content of what was taught to parents in treatment appeared to be largely consistent with the content of sessions delivered to parents of children of all ages, some of it replicating the content of adolescent sessions (e.g. psychoeducation, graded exposure), and other elements focused on parents learning how best to support their adolescent (e.g. through contingency management). One study (Siqueland et al. 2005) worked with families to directly address parental beliefs about anxiety, overprotection, and psychological control, which have been shown to be associated with adolescent anxiety symptoms/disorders (e.g. Waite et al. 2014) and to help adolescents become more autonomous. Interestingly, although the parents appeared to value this intervention, the outcomes following this treatment were not significantly different to the (adolescent only) CBT condition. Nor did the adolescents in either treatment perceive any changes in their parents’ behaviours, including psychological control and acceptance, from pre- to post-treatment. This raises the question of whether changing particular parental responses is ineffective in achieving improved adolescent outcomes, or whether the particular therapeutic techniques were ineffective in changing parental responses. We would suggest that rather than conducting more trials to compare broad and varying approaches to parent involvement, the field would benefit from a combination of dismantling and experimental studies to address these key questions.

Beyond the clinical effectiveness of parental involvement, it will also be important to understand other factors, such as the preferences of adolescents and their parents regarding parental involvement. None of the studies reported on the adolescent’s satisfaction with parental involvement. However, within the parent data, there were some indications of parents being more satisfied when involved in treatment. Waite et al. (2019) found higher levels of treatment satisfaction among parents who had completed parent sessions than those who had not, and Siqueland et al. (2005) reported that parents who did not receive the attachment-based family therapy intervention reported disappointment at the lack of parental involvement, and those who did rated this as the most important or satisfying aspect of treatment. However, there is likely to be variability in parents’ views and experiences; data from a qualitative study with parents of children and adolescents who had not responded to CBT reflected some of the challenges for parents in being involved in treatment, including lacking the time and energy required to support their child with the treatment (Lundkvist-Houndoumadi et al. 2016).

Even if there is a potential benefit to including parents in treatment, if this is being done through additional or parallel sessions, there is a question about whether the additional cost of treatment delivery can be justified. None of the studies included health economics measures in order to be able to determine the cost as well as clinical effectiveness. Waite et al. (2019) found that adding therapist-supported internet parent sessions did not improve clinical outcomes, but also that parents generally had some level of involvement in the adolescent’s treatment even if they were not completing the parent sessions (e.g. discussing the sessions with the adolescent, seeing some of the content from the adolescent’s sessions). Thus, it may not be necessary for services to dedicate resources to delivering additional input to all parents if many parents have some level of involvement regardless and are happy with that. However, this study also found that significantly fewer adolescents required a referral for further treatment when parents had completed sessions, perhaps suggesting some longer-term cost-benefits from parents being more formally involved. Including health economics measures post-treatment and at follow-up time points will be critical moving forwards. It will also be critical to better establish for whom parental involvement may be helpful (e.g. where parents are keen for guidance or in circumstances where parental beliefs and behaviours appear to be getting in the way of the adolescent’s progress in treatment (Leigh and Clark 2019)), for whom it may not be necessary (e.g. where parents are able to support treatment progress without direct guidance), and for whom it may be critical (e.g. in contexts where young people do not want to or are not able to participate in treatment). Answers to many of these questions will only come from a better understanding of how parental responses may reinforce or reduce anxiety problems in adolescents (specifically), and in what circumstances. Ultimately, we do not anticipate that the key to potential benefits of parental involvement will be based on the format or number of sessions, but it will be about whether treatment successfully changes maintenance mechanisms that prevent adolescents from overcoming problems with anxiety.

### Strengths and Limitations

This review directly addresses the longstanding criticism that the existing literature on anxiety disorders has neglected the adolescent developmental period specifically (Kendall and Ollendick 2005). The systematic nature of the review ensured a rigorous approach, and the use of a quality assessment tool enhanced the critical evaluation of the findings. Nevertheless, a number of limitations of this review must be considered. Only three studies specified a primary outcome measure, and by using a number of outcome measures without defining the primary measure potentially increases the risk of false-positive errors from multiple tests and risks inflating the effects of treatment. We made the decision to extract information relating to parental involvement from the individual papers rather than from treatment protocols because many papers did not report using published protocols, protocols were unavailable or may have been adapted for the purposes of the study. However, as the focus of the studies was on the adolescents’ treatment, descriptions of how parents were involved were often relatively brief and not always clearly specified. It is also possible that we missed some studies altogether due to a lack of reporting of parental involvement. We coded papers for the presence or absence of specific treatment components. This meant that, for example, where a paper described how the parents would ‘learn techniques to decrease their child’s avoidance’ (Masia-Warner et al. 2005), although this may have included graded exposure, it was not coded as such. Finally, the poor reporting in the majority of studies regarding recruitment processes, gender, socio-economic status and ethnicity limits the generalisability of the findings.

#### Conclusion

This review highlights that parents are commonly included in the treatment of anxiety disorders for adolescents in a variety of formats, for different durations and with varying content. Given such wide variation in how parents are involved and with only one study directly comparing outcomes with and without additional parent sessions, at this point in time it is not possible to determine the contribution of parental involvement to treatment outcomes for adolescents. We urgently need to identify whether, how, and in what contexts parents should be involved in the treatment of adolescents with anxiety disorders in the future through experimental research, dismantling studies, and efficacy trials specifically designed to address these questions.

## Electronic supplementary material

Below is the link to the electronic supplementary material.

Electronic supplementary material 1 (DOCX 48 kb)
