# Supplementary material for: A Systematic Review of Parental Involvement in Cognitive Behavioural Therapy for Adolescent Anxiety Disorders
Source: Clin Child Fam Psychol Rev. 2020 Aug 30;23(4):483–509. doi: 10.1007/s10567-020-00324-2 (PMC7585571; doi:10.1007/s10567-020-00324-2)
Supplement: Supplementary file 1 — Electronic supplementary material 1 (DOCX 48 kb) [file 10567_2020_324_MOESM1_ESM.docx]

Online Resource 1: Quality appraisal table

| Author (Year) |  | Albano et al. (1995) | Christon et al. (2012) | Elkins et al. (2016) | Heard et al. (1992) | Hoffman & Mattis (2000) | Leyfer et al. (2018) | Ollendick (1995) | Pincus et al. (2010) | Anderson et al. (1998) | Baer & Garland (2005) | Legerstee et al. (2008) | Leigh & Clark (2016) | Masia-Warner et al. (2005) | Masia-Warner et al. (2007) | Masia-Warner et al. (2016) | Nordh et al. (2017) | Spence et al. (2008) | Spence et al. (2011) | Waite et al. (2019) | Kendall & Barmish (2007) | Siqueland et al. (2005) Phase I | Siqueland et al. (2005) Phase II | Stjerneklar et al. (2018) | Wuthrich et al. (2012) |
| --- | --- | --- | --- | --- | --- | --- | --- | --- | --- | --- | --- | --- | --- | --- | --- | --- | --- | --- | --- | --- | --- | --- | --- | --- | --- |
| Reporting | Aims | 1 | 1 | 1 | 1 | 1 | 1 | 1 | 1 | 1 | 1 | 1 | 1 | 1 | 1 | 1 | 1 | 1 | 1 | 1 | 0 | 1 | 1 | 1 | 1 |
|  | Outcomes | 1 | 0 | 1 | 1 | 0 | 1 | 1 | 1 | 0 | 1 | 1 | 1 | 1 | 1 | 1 | 1 | 1 | 1 | 1 | 0 | 1 | 1 | 1 | 1 |
|  | Participants | 1 | 1 | 1 | 1 | 1 | 1 | 1 | 1 | 1 | 1 | 1 | 1 | 1 | 1 | 1 | 1 | 1 | 1 | 1 | 1 | 1 | 1 | 1 | 1 |
|  | Interventions | 1 | 1 | 1 | 1 | 1 | 1 | 1 | 1 | 1 | 1 | 0 | 1 | 1 | 1 | 1 | 1 | 1 | 1 | 1 | 1 | 1 | 1 | 1 | 1 |
|  | Parental involvement | 1 | 1 | 0 | 1 | 0 | 1 | 0 | 1 | 1 | 0 | 0 | 0 | 1 | 1 | 0 | 1 | 1 | 1 | 1 | 1 | 1 | 1 | 1 | 1 |
|  | Confounders | 0 | 2 | 2 | 1 | 0 | 2 | 2 | 1 | 0 | 2 | 2 | 2 | 2 | 2 | 2 | 0 | 1 | 1 | 2 | 1 | 1 | 1 | 2 | 1 |
|  | Main findings | 1 | 0 | 1 | 1 | 1 | 1 | 1 | 1 | 1 | 1 | 1 | 1 | 1 | 1 | 1 | 1 | 1 | 1 | 1 | 0 | 1 | 1 | 1 | 1 |
|  | Random variability | 0 | 0 | 1 | 0 | 0 | 1 | 1 | 1 | 0 | 1 | 1 | 1 | 1 | 1 | 1 | 1 | 0 | 1 | 1 | 0 | 1 | 1 | 1 | 1 |
|  | Adverse events | 0 | 0 | 0 | 0 | 0 | 1 | 0 | 0 | 0 | 0 | 0 | 0 | 0 | 0 | 0 | 1 | 0 | 0 | 0 | 0 | 0 | 0 | 0 | 0 |
|  | Dropouts | 1 | 1 | 0 | 1 | 1 | 1 | 1 | 1 | 1 | 1 | 1 | 1 | 0 | 0 | 1 | 1 | 1 | 0 | 1 | 1 | 1 | 0 | 1 | 1 |
|  | Probability values | 0 | 0 | 1 | 0 | 0 | 1 | 0 | 0 | 0 | 1 | 1 | 0 | 1 | 1 | 0 | 1 | 0 | 1 | 1 | 0 | 0 | 1 | 0 | 1 |
| External validity | Sampling representative | 1 | 0 | 0 | 0 | 0 | 1 | 0 | 1 | 0 | 1 | 1 | 0 | 1 | 1 | 1 | 1 | 0 | 1 | 1 | 0 | 1 | 1 | 0 | 0 |
|  | Participants representative | 1 | 0 | 0 | 0 | 0 | 0 | 0 | 0 | 0 | 0 | 0 | 0 | 0 | 0 | 0 | 0 | 0 | 0 | 0 | 0 | 0 | 0 | 0 | 0 |
|  | Treatment representation | 0 | 0 | 0 | 1 | 0 | 0 | 0 | 0 | 0 | 1 | 1 | 0 | 0 | 0 | 0 | 0 | 0 | 0 | 1 | 1 | 0 | 0 | 0 | 0 |
| Internal validity | Blind to intervention | 0 | 0 | 0 | 0 | 0 | 0 | 0 | 0 | 0 | 0 | 0 | 0 | 0 | 0 | 0 | 0 | 0 | 0 | 0 | 0 | 0 | 0 | 0 | 0 |
|  | Blind to outcome measures | 1 | 0 | 1 | 0 | 0 | 1 | 0 | 0 | 0 | 0 | 0 | 0 | 1 | 1 | 1 | 1 | 1 | 1 | 1 | 0 | 0 | 0 | 0 | 0 |
|  | Data dredging | 1 | 1 | 1 | 1 | 1 | 1 | 1 | 1 | 1 | 1 | 1 | 1 | 0 | 1 | 1 | 1 | 1 | 1 | 1 | 1 | 1 | 1 | 1 | 1 |
|  | Analysis adjustment | 1 | 1 | 1 | 1 | 1 | 1 | 1 | 1 | 0 | 0 | 1 | 1 | 0 | 1 | 1 | 1 | 0 | 1 | 1 | 0 | 1 | 1 | 1 | 1 |
|  | Statistical tests | 0 | 1 | 1 | 0 | 1 | 1 | 1 | 1 | 1 | 1 | 1 | 1 | 1 | 1 | 1 | 1 | 1 | 1 | 1 | 1 | 1 | 1 | 1 | 1 |
|  | Reliable compliance | 1 | 1 | 1 | 1 | 1 | 1 | 1 | 1 | 1 | 1 | 1 | 1 | 1 | 1 | 1 | 1 | 1 | 1 | 1 | 1 | 1 | 1 | 1 | 1 |
|  | Reliable outcome measures | 1 | 1 | 1 | 1 | 1 | 1 | 1 | 1 | 1 | 1 | 1 | 1 | 1 | 1 | 1 | 1 | 1 | 1 | 1 | 0 | 1 | 1 | 1 | 1 |
|  | Same population for intervention groups | 1 | 0 | 1 | 1 | 1 | 1 | 1 | 1 | 0 | 1 | 1 | 1 | 1 | 1 | 1 | 1 | 0 | 1 | 1 | 0 | 1 | 1 | 1 | 1 |
|  | Recruitment period | 0 | 0 | 0 | 0 | 1 | 0 | 0 | 0 | 0 | 0 | 1 | 0 | 0 | 0 | 0 | 1 | 0 | 1 | 1 | 0 | 0 | 0 | 0 | 1 |
|  | Randomisation | 0 | 0 | 1 | 0 | 0 | 1 | 0 | 1 | 0 | 1 | 1 | 0 | 1 | 1 | 1 | 0 | 0 | 1 | 1 | 0 | 0 | 1 | 0 | 1 |
|  | Randomisation concealment | 0 | 0 | 0 | 0 | 0 | 1 | 0 | 0 | 0 | 0 | 0 | 0 | 0 | 0 | 0 | 0 | 0 | 0 | 0 | 0 | 0 | 0 | 0 | 0 |
|  | Confounder adjustment | 0 | 0 | 1 | 0 | 0 | 1 | 0 | 1 | 0 | 0 | 1 | 0 | 1 | 1 | 1 | 1 | 0 | 1 | 1 | 0 | 1 | 0 | 0 | 1 |
|  | Losses to follow-up | 1 | 1 | 0 | 1 | 1 | 1 | 1 | 1 | 1 | 0 | 0 | 1 | 1 | 1 | 1 | 1 | 0 | 1 | 1 | 1 | 1 | 0 | 0 | 1 |
| Power | Power | 0 | 0 | 0 | 0 | 0 | 1 | 0 | 1 | 0 | 0 | 0 | 0 | 0 | 1 | 1 | 1 | 0 | 1 | 1 | 0 | 0 | 0 | 0 | 1 |
| Total Score |  | 16 | 13 | 18 | 15 | 13 | 25 | 16 | 20 | 11 | 18 | 20 | 16 | 19 | 22 | 21 | 22 | 13 | 22 | 25 | 10 | 18 | 17 | 16 | 21 |

Online Resource 2: References for Table 2

**Anxiety Disorders Interview Schedule for Children, Child and Parent Versions (ADIS-C/P)**

Albano, A. M., & Silverman, W. K. (1996). *Anxiety Disorders Interview Schedule for DSM-IV-Child Version: Clinician Manual.* Psychological Corporation.

**Attachment-based Family Therapy (ABFT)**

Adapted from:

Diamond, G. S., Reis, B. F., Diamond, G. M., Siqueland, L., & Isaacs, L. (2002). Attachment-based family therapy for depressed adolescents: A treatment development study. *Journal of the American Academy of Child & Adolescent Psychiatry, 41,* 1190-1196. doi:10.1097/00004583-200210000-00008

Diamond, G., Siqueland, L., & Diamond, G. M. (2003). Attachment-based family therapy for depressed adolescents: Programmatic treatment development. *Clinical Child and Family Psychology Review, 6,* 107-127. doi:10.1023/A:1023782510786

**Beck Anxiety Inventory (BAI)**

Beck, A. T., Epstein, N., Brown, G., & Steer, R. A. (1988). An inventory for measuring clinical anxiety: Psychometric properties. *Journal of Consulting and Clinical Psychology, 56,* 893. doi:10.1037/0022-006X.56.6.893

**BRAVE-CLINIC**

Donovan, Cl. L., Holmes, J., & Spence, S. H. (2006). *BRAVE for Teenagers: A Program for Adolescents with Anxiety.* School of Psychology, The University of Queensland.

**BRAVE-for Teenagers ONLINE**

Spence, S. H., Holmes, J., Donovan, C. L., & Kenardy, J. (2006). *BRAVE for Teenagers-ONLINE: An Internet-Based Program for Adolescents with Anxiety.* Brisbane: University of Queensland.

**Child Behaviour Checklist (CBCL)**

Achenbach, T. M., & Edelbrock, C. S. (1983). *Manual for the Child Behavior Checklist and Revised Child Behavior Profile.* Burlington: University of Vermont, Department of Psychiatry.

**CBT for Panic with Agoraphobia**

Adapted from:

Barlow, D. H., Craske, M. G., Cerny, J. A., & Klosko, J. S. (1989). Behavioral treatment of panic disorder. *Behavior therapy, 20,* 261-282. doi:10.1016/S0005-7894(89)80073-5

Öst, L. G., Westling, B. E., & Hellström, K. (1993). Applied relaxation, exposure in vivo and cognitive methods in the treatment of panic disorder with agoraphobia. *Behaviour Research and Therapy, 31,* 383-394. doi:10.1016/0005-7967(93)90095-C

**CBT for School Refusal**

Heyne, D., & Rollings, S. (2002). *School refusal (parent, adolescent and child training skills).* Blackwell Publishers, Oxford.

**CBT modified for Adolescents**

Kendall, P. C., & Hedtke, K. A. (2006). *Cognitive-behavioral therapy for anxious children: Therapist manual.*

**CBT with Parent and School Involvement**

Mansdorf, I. J., & Lukens, E. (1987). Cognitive-behavioral psychotherapy for separation anxious children exhibiting school phobia. *Journal of the American Academy of Child & Adolescent Psychiatry, 26,* 222-225. doi:10.1097/00004583-198703000-00018

**Childhood Anxiety Sensitivity Index (CASI)**

Silverman, W. K., Fleisig, W., Rabian, B., & Peterson, R. A. (1991). Childhood anxiety sensitivity index. *Journal of Clinical Child and Adolescent Psychology, 20,* 162-168. doi:10.1207/s15374424jccp2002_7

**Children’s Depression Inventory (CDI)**

Kovacs, M., & Beck, A. T. (1977). An empirical-clinical approach toward a definition of childhood depression. *Depression in childhood: Diagnosis, treatment, and conceptual models*, 1-25.

**Children’s Global Assessment Scale (CGAS)**

Shaffer, D., Gould, M. S., Brasic, J., Ambrosini, P., Fisher, P., Bird, H., & Aluwahlia, S. (1983). A children's global assessment scale (CGAS). *Archives of General psychiatry, 40,* 1228-1231. doi:10.1001/archpsyc.1983.01790100074010

**Children’s Report of Parenting Behaviour Inventory (CRPBI)**

Schuldermann, E., & Schuldermann, S. (1970). Replicability of the factor in children’s report of parent behavior. *Journal of Psychology, 76,* 239-249. doi:10.1080/00223980.1970.9916845

**Chilled Out**

Wuthrich, V. M., Cunningham, M. J., & Rapee, R. M. (2013). Cool teens: A computerized intervention for anxious adolescents. *The Wiley‐Blackwell Handbook of The Treatment of Childhood and Adolescent Anxiety,* 249-274. doi:10.1002/9781118315088.ch12

**Chilled Out Mentor Companion**

Wuthrich, V. M. (2014). Chilled Out Mentor Companion. *Centre for Emotional Health, Macquarie University, Sydney.*

**Clinical Global Impression-Severity (CGI-S)**

Guy, W. (1976). Clinical global impressions scale: ECDEU assessment manual for psychopharmacology. *US Department of Health, Education, and Welfare publication (AMD) 76,* 221-227. doi:10.1037/e591322011-001

**Cognitive-Behavioural Group Treatment for Adolescents (CBGT-A)**

Albano, A. M., Marten, P. A., & Holt, C. S. (1991). Cognitive-behavioural group treatment of adolescent social phobia: Therapist’s manual. *Unpublished manuscript, State University of New York, Albany.*

**Cognitive Therapy for Social Anxiety Disorder (CT-SAD)**

Clark, D. M., & Wells, A. (1995). A cognitive model of social phobia. *Social Phobia: Diagnosis, Assessment, and Treatment, 41,* 22-23.

**Composite International Diagnostic Interview (CIDI)**

World Health Organization. (1990). *Composite International Diagnostic Interview (CIDI), version 2.1.* Geneva: World Health Organization.

**Cool Teens**

Cunningham, M., Rapee, R., & Lyneham, H. (2006). The Cool Teens CD-ROM: A multimedia self-help program for adolescents with anxiety. *Youth Studies Australia, 25,* 50.

**Coping Cat**

Kendall, P. C. (1990). *Coping Cat Manual.* Ardmore, PA: Workbook.

**Dyadic Adjustment Scale (DAS)**

Spanier, G. B. (1976). Measuring dyadic adjustment: New scales for assessing the quality of marriage and similar dyanxiety disorders. *Journal of Marriage and the Family,* 15-28. doi:10.2307/350547

**FRIENDS**

Barrett, P., Turner, C., & Lowry-Webster, H. (2000). *Friends for Children Group Leader's Manual.* Australian Academic Press.

**Hamilton Anxiety Rating Scale (HAM-A)**

Hamilton, M. A. (1959). The assessment of anxiety states by rating. *British Journal of Medical Psychology, 32,* 50-55. doi:10.1111/j.2044-8341.1959.tb00467.x

**Kiddie Schedule for Affective Disorders and Schizophrenia-Present and Lifetime Version (K-SANXIETY DISORDERS-PL)**

Kaufman, J., Birmaher, B., Brent, D., Rao, U. M. A., Flynn, C., Moreci, P., ... & Ryan, N. (1997). Schedule for affective disorders and schizophrenia for school-age children-present and lifetime version (K-SANXIETY DISORDERS-PL): Initial reliability and validity data. *Journal of the American Academy of Child & Adolescent Psychiatry, 36,* 980-988. doi:10.1097/00004583-199707000-00021

**Liebowitz Social Anxiety Scale for Children and Adolescents (LSAS-CA)**

Masia-Warner, C., Storch, E. A., Pincus, D. B., Klein, R. G., Heimberg, R. G., & Liebowitz, M. R. (2003). The Liebowitz social anxiety scale for children and adolescents: An initial psychometric investigation. *Journal of the American Academy of Child & Adolescent Psychiatry, 42,* 1076-1084. doi:10.1097/01.CHI.0000070249.24125.89

**Modular Approach to Therapy for Children with Anxiety, Depression, Trauma, or Conduct Problems (MATCH)**

Chorpita, B. F., & Weisz, J. R. (2009). *Modular Approach to Therapy for Children with Anxiety, Depression, Trauma, or Conduct Problems (MATCH-ADTC).* Satellite Beach, FL: PracticeWise.

**Multidimensional Anxiety Scale for Children (MASC)**

March, J. S., Parker, J. D., Sullivan, K., Stallings, P., & Conners, C. K. (1997). The Multidimensional Anxiety Scale for Children (MASC): Factor structure, reliability, and validity. *Journal of the American Academy of Child & Adolescent Psychiatry, 36,* 554-565. doi:10.1097/00004583-199704000-00019

**Panic Control Treatment for Adolescents (PCT-A)**

Angelosante, A. G., Pincus, D. B., Whitton, S. W., Cheron, D., & Pian, J. (2009). Implementation of an intensive treatment protocol for adolescents with panic disorder and agoraphobia. *Cognitive and Behavioral Practice, 16,* 345-357. doi:10.1016/j.cbpra.2009.03.002

Barlow, D. H., Craske, M. G., Cerny, J. A., & Klosko, J. S. (1989). Behavioral treatment of panic disorder. *Behavior Therapy, 20,* 261-282. doi:10.1016/S0005-7894(89)80073-5

Pincus, D. B., Ehrenreich, J. T., & Mattis, S. G. (2008). *Mastery of Anxiety and Panic for Adolescents Riding the Wave, Therapist Guide.* Oxford University Press.

**Panic Disorder Severity Scale for Children (PDSS-C)**

Elkins, R. M., Pincus, D. B., & Comer, J. S. (2014). A psychometric evaluation of the Panic Disorder Severity Scale for children and adolescents. *Psychological Assessment, 26,* 609. doi:10.1037/a0035283.

**Revised Child Anxiety and Depression Scale-Child / Parent form (RCANXIETY DISORDERS-C/P)**

Chorpita, B. F., Moffitt, C. E., & Gray, J. (2005). Psychometric properties of the Revised Child Anxiety and Depression Scale in a clinical sample. *Behaviour Research and Therapy, 43,* 309-322. doi:10.1016/j.brat.2004.02.004

**Revised Children’s Manifest Anxiety Scale (RCMAS)**

Reynolds, C. R., & Richmond, B. O. (1978). What I think and feel: A revised measure of children's manifest anxiety. *Journal of Abnormal Child Psychology, 6,* 271-280. doi:10.1007/BF00919131

**Skills for Academic and Social Success (SASS)**

Masia, C., Beidel, D. C., Albano, A. M., Rapee, R. M., Turner, S. M., Morris, T. L., & Klein, R. G. (1999). *Skills for Academic and Social Success.* New York: Child Study Center.

**Social Effectiveness Therapy for Children and Adolescents (SET-C)**

Beidel, D. C., Turner, S. M., & Morris, T. L. (1998). Social effectiveness therapy for children: A treatment manual. *Unpublished manuscript, Medical University of South Carolina.*

**Social Phobia Weekly Summary Scale (SPWSS)**

Clark, D. M., Ehlers, A., McManus, F., Hackmann, A., Fennell, M., Campbell, H., ... & Louis, B. (2003). Cognitive therapy versus fluoxetine in generalized social phobia: A randomized placebo-controlled trial. *Journal of Consulting and Clinical Psychology, 71,* 1058. doi:10.1037/0022-006X.71.6.1058
